# Foliar spraying with zinc oxide nanoparticles enhances the anti-osteoporotic efficacy of the fruit extracts of *Silybum marianum* L. by stimulating silybin production

**DOI:** 10.3389/fpls.2024.1421485

**Published:** 2025-01-07

**Authors:** Bedoor Fahad Almulhim, Fadia El Sherif, Nancy S. Younis, Yamen Safwat, Salah Khattab

**Affiliations:** ^1^ Department of Biological Sciences, College of Science, King Faisal University, Al-Ahsa, Saudi Arabia; ^2^ Department of Horticulture, Faculty of Agriculture, Suez Canal University, Ismalia, Egypt; ^3^ Department of Pharmaceutical Sciences, College of Clinical Pharmacy, King Faisal University, Al-Ahsa, Saudi Arabia; ^4^ Department of Orthopedic Surgery, Zagazig University, Zagazig, Egypt

**Keywords:** zinc dioxide, nanoparticles, *Silybum marianum*, silybin, chalcone synthase, dexamethasone, osteoporosis

## Abstract

**Introduction:**

*Silybum marianum* is a medicinal plant that produces silymarin, which has been demonstrated to possess antiviral, anti-neurodegenerative, and anticancer activities. Silybin (A+B) are two major hepatoprotective flavonolignans produced predominantly in *S. marianum* fruits. Several attempts have been made to increase the synthesis of silymarin, or its primary components, silybin (A+B). Zinc oxide nanoparticles (ZnO-NPs) are considered a highly efficient Zn source widely used to promote crop development and productivity.

**Methods:**

In this study, we aimed to investigate the effects of the foliar application of ZnO-NPs on the growth, yield, photosynthetic pigment content, silybin (A+B) content, and the expression of the chalcone synthase (*CHS*) gene in *S. marianum* plants. Different concentrations of ZnO-NPs were administered as foliar sprays to *S. marianum* plants growing in greenhouse conditions. Furthermore, we evaluated the anti-osteoporotic efficacy of the corresponding fruit extract against dexamethasone (Dex)-induced osteoporosis.

**Results and discussion:**

Foliar treatment at all ZnO-NP concentrations increased the amounts of bioactive components of silybin (A+B), which enhanced the growth and yield of *S. marianum* plants while increasing the levels of N, P, K, and Zn in their leaves, roots, and fruits; the levels of photosynthetic pigments in their leaves; and silybin (A+B) content in their fruits, thereby increasing the medicinal value of *S. marianum*. The highest gains were observed in plants sprayed with the highest ZnO-NP concentration (20.0 mg/L). In addition, gene expression studies revealed that ZnO-NPs stimulated silybin (A+B) production by activating *CHS* genes. The administration of *S. marianum* extracts to Dex-administered rats increased osteoblast and bone formation while inhibiting osteoclast and bone resorption, thereby protecting the animals against Dex-induced osteoporosis.

## 1 Introduction

The commercial cultivation of certain medicinal plants is necessary to meet the rising demands of pharmaceutical manufacturing and traditional therapeutic systems. Increased plant growth and development yield with medicinal value have been documented as benefits of zinc oxide nanoparticles (ZnO-NPs) (El Sherif et al., 2023; Khattab et al., 2023; Takahashi et al., 2010). The foliar spraying of ZnO-NPs can have beneficial or detrimental impacts on plant growth and yield compared with that of the ionic form (Zn^2+^) of the correlative metal and microparticles or macroparticles (Awan et al., 2021). ZnO-NPs provide a highly efficient Zn source because of their NP features that enable them to cross the plant cell membrane, transport substances into cells, and incorporate them into metabolic processes (Akanbi-Gada et al., 2019; Miliauskienė et al., 2022). They were employed to boost plant productivity and yield in a variety of species (Al Jabri et al., 2022; Faizan et al., 2021; Adil et al., 2022), as zinc is responsible for plant defense and cell-membrane stabilization, supports protein translation, regulates extensive membrane activities for cell elongation, and provides a defensive mechanism against environmental (Rudani et al., 2018).

Milk thistle (*Silybum marianum* L.) is a spiny winter annual or biennial herb, which originated in the Middle East and Mediterranean regions and belongs to the Asteraceae family. The species is widely distributed worldwide, existing in native and allochthonous forms. This unique plant is highly adaptive and flourishes in various environments (Geremew et al., 2023). The pharmacological actions of *S. marianum* are linked to silymarin, a bioactive molecule produced by seeds and fruits and used to treat several liver disorders. Silymarin is a stereoisomeric flavonoid complex composed of silybin, isosilybin, silydianin, silychristin, and the precursor of silychristin, taxifolin (Page and Feller, 2015). The hepatoprotective properties of silymarin are linked to natural silybin, a mix of silybin A and B, which exhibits neuroprotective activity in Alzheimer’s treatment by altering brain-derived neuroprotective factors, central regulators, physiological process regulators, and other causative agents associated with neurodegeneration, neurotoxicity, and cancer (Claus et al., 2013). In many plant species, chalcone synthase (CHS), an allosteric enzyme, is crucial for the production of flavonoids and is responsible for the synthesis of flavonolignans in *S. marianum*. The expression of *CHS-1*, *CHS-2*, and *CHS-3* genes is responsible for building up silymarin content in *S. marianum* (El Sherif et al., 2020; García-López et al., 2019; Wang et al., 2023; Maher et al., 2023).

Humans have used plants and their extracts as natural remedies for managing diverse diseases. Therefore, plants have been under extensive investigation for their biological activity. These diverse plants may offer a way to improve the bone structure and function. Osteoporosis, a common systemic bone disease, is associated with bone erosion, damaged bone internal architecture, and reduced bone modeling and remodeling activities with bone mass density, leading to an increased susceptibility to fractures (Sanjari et al., 2015). Osteoporosis is categorized as primary osteoporosis in postmenopausal, elderly populations and may arise spontaneously (Alkuwayti et al., 2020). However, secondary osteoporosis occurs from pathologic or injurious conditions, or it may be drug-induced, e.g., consuming glucocorticoids (GCs) for a long term in a high dose may lead to secondary osteoporosis (Hachemi et al., 2018). GCs, the gold standard drug for managing diverse inflammatory and autoimmune disorders, may induce osteoporosis by altering the equilibrium between the rate of degradation of pathologic bone cells and the rate of formation of new bone cells (Kim et al., 2013). Current pharmacotherapies for osteoporosis, including antiresorptive agents (bisphosphonates), reduce the rate of resorption of osteoclasts and are accompanied by severe adverse effects, including jaw osteonecrosis and renal toxicity (Saleh et al., 2022). Therefore, searching for superior, efficient, and safe substitutes for managing osteoporosis is imperative.

In this study, we explored the positive outcomes of different concentrations of ZnO-NPs on the growth, productivity, photosynthetic pigment quality, and silybin (A+B) contents of *S. marianum* plants. The expression of *CHS* genes was also examined to establish a connection between silybin (A+B) synthesis and *CHS* gene transcription. Furthermore, *S. marianum* fruit extract obtained from the fruits of plants sprayed with ZnO-NPs has been evaluated for its anti-osteoporotic efficacy against dexamethasone (Dex)-induced osteoporosis and the underlying molecular mechanisms, primarily through the participation of Runx2, receptor activator of nuclear factor κB (NF-κB) ligand/osteoprotegerin (RANKL/OPG) pathways, and oxidative stress.

## 2 Materials and methods

### 2.1 *Silybum marianum* greenhouse cultivation

*Silybum marianum* seeds were obtained from the Agricultural-Veterinarian Training and Research Station, King Faisal University, Al-Ahsa, Saudi Arabia, and cultivated in a greenhouse in the same place from 1 October 2022 to 31 July 2023. The experiment followed a completely randomized design with 20 repetitions performed under the previously reported greenhouse conditions, using the same soil features, irrigation conditions, and growing techniques (El Sherif et al., 2023). The plants of different treatment groups were sprayed with four different concentrations of ZnO-NPs, i.e., 0.0 mg/L (control, distilled water), 5.0 mg/L, 10.0 mg/L, and 20.0 mg/L (El Sherif et al., 2023) every 2 months: 1 December 2022, 1 February 2023, and 1 April 2023, during the season favorable for the vegetative growth stage of *S. marianum*. Five drops of dimethyl sulfoxide were added to ZnO-NPs, and this mix was then diluted with distilled water to achieve the desired treatment concentrations. ZnO-NPs were sprayed around 8 am. Approximately 50 mL of ZnO-NP solutions (5.0 mg/L, 10.0 mg/L, and 20.0 mg/L) or distilled water (control) were sprayed onto every plant (aerial parts). ZnO-NP powder (Cat. No. 677450) with an average particle size of 50 nm was purchased from Sigma-Aldrich (Germany). The irrigation conditions and soil chemical properties, including its mineral content, used during the experiment are shown in **Supplementary Tables S1** and **S2**. Irrigation, fertilization, and weeding were performed as recommended.

### 2.2 Evaluation of *S. marianum* growth and yield characteristics

After sowing, the data regarding the growth rate of 10 randomly selected plant from each treatment group were recorded at 270 days. The data statistics comprised plant height (cm), number of branches and leaves (n), and the dry weight of aerial parts and roots (g). The mature fruits of plants from each treatment group were regularly assembled and dried at room temperature. The dried fruit mass and number of capitula (n) for each plant were noted.

### 2.3 Quantitative evaluation of K, N, P, and Zn contents in *S. marianum* leaves, roots, and fruits and the quantification of photosynthetic pigment contents in *S. marianum* leaves

At 270 days post-sowing, 10 non-specified plants were selected and harvested from each treatment group. The levels of N, P, K, and Zn in leaves, roots, and fruits and the levels of photosynthetic pigments in leaves were estimated, as described earlier (El Sherif et al., 2023; Khattab et al., 2023; Peng et al., 2021).

### 2.4 Quantification of silybin (A+B) contents in *S. marianum* fruits using high-performance liquid chromatography

#### 2.4.1 Instrumentation

The 2690 Alliance HPLC system (Waters, Milford, MA, USA) was used for quantifying silybin (A+B) contents in fruits harvested from 10 *S. marianum* plants against natural silybin (A+B) and a standard (Santa Cruz Biotechnology, Dallas, TX, USA). The separation module consisted of a Kromasil C18 column (4.6 mm × 150 mm, 5 µm) and a 996 photodiode array detector (Waters, USA). The preparation of the silybin (A+B) standard and methanolic fruit extracts of *S. marianum* and their chromatographic conditions have been previously described (El Sherif et al., 2023).

#### 2.4.2 Preparation of the stock solution

Furthermore, the silybin (A+B) stock solution of 1 mg/mL concentration in methanol was prepared using the authentic silybin (A+B) standard (Cat. No. sc-473918) purchased from Santa Cruz Biotechnology Inc., USA. Five serial dilutions were prepared at the concentrations of 750 μg/mL, 500 μg/mL, 250 μg/mL, 100 μg/mL, and 50 μg/mL. Each dilution was filtered using a 0.22-μm syringe filter before 10 μL of each dilution was separately injected into the high-performance liquid chromatography (HPLC) system.

#### 2.4.3 Preparation of *S. marianum* fruit extract

A known weight of each air-dried fruit sample was taken into a conical flask, and then 50 mL of methanol was added to the flask. The samples were then sonicated for 30 min before being kept in the dark for 24 h. Subsequently, the suspension was filtered and the filtrate was collected and kept aside. The extraction process was repeated by adding another 50 mL of fresh methanol to the residue from the filtration. This extraction process was repeated for three successive days, and the filtrate collected from these days was evaporated on a rotary evaporator maintained at 40°C to obtain the dry residue for each air-dried sample. Subsequently, a known weight of the dried residue was dissolved in 5 mL of ethanol before each solution was filtered using a 0.22-μm syringe filter. Approximately 10 μL of each sample was then injected into the HPLC system.

#### 2.4.4 HPLC analysis conditions

Subsequently, HPLC was performed at an ambient temperature using the Kromasil C18 column (4.6 mm × 150 mm, 5 μm). The gradient elution was accomplished by the utilization of two solvents: solvent A (water: methanol: phosphoric acid, 80:20:0.5, v:v:v) and solvent B (methanol: water: phosphoric acid, 80:20:0.5, v:v:v). The flow rate of the mobile phase was maintained at 1 mL/min, whereas the eluate was detected at 288 nm.

### 2.5 Evaluation of the expression of *CHS 1*, *2*, and *3* genes using quantitative real-time polymerase chain reaction

Gene-specific primers (**Supplementary Table S3**) were used to examine the expression of *CHS 1*, *2*, and *3* genes in the petals of six *S. marianum* plants. The forward and reverse primers for the three target genes *CHS1*, *CHS2*, and *CHS3*, and a reference gene, nicotinamide adenine dinucleotide dehydrogenase, were designed using GenBank gene sequences. The quantitative real-time polymerase chain reaction (qPCR) was performed on the Applied Biosystems 7500 Real-time PCR system (Thermo Fisher Scientific, Waltham, MA, USA). The expression of *CHS* genes was measured using the 2^−ΔΔCT^ technique (Tao et al., 2022), which normalized the target gene expression with that of the reference gene. The mean expressions of the three *CHS* genes in each treatment group were computed using six biological samples, each with two technical replicates. The relative fold expressions of *CHS1*, *CHS2*, and *CHS3* genes in each ZnO-NP treatment group were determined and compared with the expression of the corresponding genes in the control treatment (distilled water) group.

### 2.6 Animals and ethical approval

There were 56 adult Sprague–Dawley male rats (age: 4–5 weeks; body weight: 150 g–170 g) obtained from the Experimental Animal Research Centre, King Saud University. Laboratory food and water were provided ad libitum to all tested animals in the ventilated cage system (20.3°C–23.1°C, 12-h light/dark). ARRIVE (Animal Research Reporting of *In Vivo* Experiments) guidelines were complied with throughout the experiment. All animal experiments performed in this study were approved by the Institutional Animal Care and Use Committee of the King Faisal University (approval number: KFU-REC-2023-DEC-ETHICS1872).

### 2.7 Experimental design

Rats were distributed into five groups (n=6) after a week of acclimatization. The first group comprised the control group, which received saline daily for 8 weeks. The second group comprised the Dex group, which represented the Dex-induced osteoporosis group wherein animals received dexamethasone (Dex) daily (1 mg/kg, i.p.) for 6 weeks (Takahashi et al., 2010). The other three groups were the Sily-100+Dex, Sily-200+Dex, and Alen+Dex groups wherein animals were administered *S. marianum* extracts (Sily-100: 100 mg/kg; Sily-200: 200 mg/kg) (Kim et al., 2012b) or alendronate (0.5 ml/100 g) (Kim et al., 2012a) daily through gavage for the 2 weeks before Dex administration then continued with Dex administration for another 6 weeks.

### 2.8 Blood collection and bone homogenate preparation

After the experiment, the animals were restricted from feeding overnight and anesthetized with isoflurane. The blood samples of the tested animals were kept aside for 15 min for clot formation at room temperature. For serum collection, blood specimens were centrifuged for 15 min at 3,000 rpm and a temperature of approximately 4°C. After centrifugation, the supernatant blood serum was collected and stored at 20°C for further use. Subsequently, the animals were sacrificed, and their femur bone was collected. The connective tissues and muscles attached to the femurs were removed. The left femurs of all tested animals were washed with cold saline and crushed using liquid nitrogen.

The crushed femurs were divided into four portions, of which two portions were kept at an intense 80°C temperature. One separate portion was used to evaluate the expression levels of the examined genes. The remaining portion was homogenized using nine volumes of phosphate-buffered cold saline (0.1 M, pH 7.4). Subsequently, it was subjected to centrifugation at 4,000 rpm for 15 min at 4°C for biochemical evaluation, following which the top layer was picked up and kept at 80°C.

### 2.9 Bone density test

Bone mineral content (BMC) and bone area ratio were compared to estimate bone mineral density (BMD). Femoral BMD (g/cm^2^) and BMC g were measured through bone densitometry.

### 2.10 Biochemical determination of the serum and bone biomarkers of osteoporosis

Commercial diagnostic kits were used to measure the serum levels of Ca and inorganic P (mg/dL), provided by BioSystems SA Costa Brava (Barcelona, Spain), following the manufacturer’s instructions. The levels or activity of serum biomarkers, including carboxy-terminal type I collagen cross-links (CTX-I) (Cat. No.: abx256844; Abbexa Ltd., Cambridge, United Kingdom), tartrate-resistant acid phosphatase 5b (TRAP5b) (Cat. No.: E-EL-R0939; Elabscience^®^ (TX, USA), and bone alkaline phosphatase (bALP) (Cat. No.: MBS265845; MyBioSource Inc. (CA, USA), were estimated using ELISA kits.

In the bone homogenate supernatant, oxidative stress and lipid peroxidation indices were estimated using the following reagents and kits: malondialdehyde (MDA, µmol/mg protein) (Cat. No.: E-BC-K025-M), bone glutathione peroxidase (GPx) kit (Cat. No.: MBS3809170), and glutathione (GSH, µmol/mg protein) content kit (Cat. No.: MBS265966) purchased from MyBioSource Inc., USA, and bone nitric oxide (NO, µmol/g protein) kit (Cat. No.: E-BC-K035-S) and superoxide dismutase (SOD, U/mg protein) activity kit (Cat. No.: E-BC-K022-M) purchased from Elabscience^®^, USA. In addition, we evaluated the levels of pro- and anti-apoptotic BCL2-associated X protein (BAX; Cat. No.: MBS8804675) and B-cell lymphoma 2 (BCL2; Cat. No.: MBS704498), the indicators of apoptosis in bone tissues, using an ELISA kit obtained from MyBioSource Inc. USA.

### 2.11 Gene expression evaluation using qPCR

The expression levels of the genes *RANKL*, *OPG*, *Runx2*, and osteocalcin (*OC*) were determined by qPCR. RNA was extracted using TRIzol, following the manufacturer’s instructions (Invitrogen, Carlsbad, CA, USA). cDNA was synthesized using SuperScript II Reverse Transcriptase (Invitrogen, USA) and amplified using SYBR Green PCR Master Mix (Applied Biosystems, Foster City, CA, USA). The samples were analyzed in triplicates, and β actin was used as the housekeeping gene. The C_t_ values were measured, and the fold changes in gene expression were analyzed using the 2^−ΔΔCt^ method after normalizing the expression levels of all target genes to that of the reference gene glyceraldehyde-3-phosphate dehydrogenase (*GAPDH*). Primer sequences used in this study have been previously described (28) and were as follows: *Runx2*, forward: 5′-GACTGTGGTTACCGTCATGGC-3′ and reverse: 5′-ACTTGGTTTTTCATAAC AGCGGA-3′; *OPG*, forward: 5′-AAAGCACCCTGTA GAAAACA-3′ and reverse: 5′-CCGTTTTATCCTCTCTA CACTC-3′; *RANKL*, forward: 5′-TATGATG GAAGGCTCATGGT-3′ and reverse: 5′-TGTCCTGAAC TTTGAAAGCC-3′; *OC*, forward: 5′-CTAGCGGACCACATTGGCTT-3′ and reverse: 5′-GCTGTGCCGTCCATACTTTC-3′; and *GAPDH*, forward: 5′-TGGCCTTCCGTGTTCC TAC-3′ and reverse: 5′-GAGTTGCTGTTGAAGTCGCA-3′.

### 2.12 Statistical analyses

Data are expressed as mean ± standard deviation (SD). For the *post-hoc* test, a one-way analysis of variance was performed, followed by the Tukey–Kramer test. The significance was tested at a probability level of *p <*0.05. GraphPad software (version 8; San Diego, CA, USA) was used for statistical analyses.

## 3 Results

### 3.1 Plant growth and yield

Plant height, leaf number, branch number, and the dry weights of the aerial parts and roots of *S. marianum* plants under ZnO-NP treatment are presented in **Table 1**. A comparative analysis of the effects of the ZnO-NP foliar spray treatment with those of the control treatment on the abovementioned parameters revealed that the values of all the studied parameters gradually enhanced in the ZnO-NP-treated plants. Significantly higher leaf numbers, branch numbers, and dry weights of aerial parts and roots were observed in plants treated with 20.0 mg/L ZnO-NPs than in plants subjected to the control treatment. In contrast, the foliar application of ZnO-NPs at 5.0 mg/L concentration resulted in the greatest plant height.

**Table 1:** Effects of different ZnO-NP concentrations on plant height, number of leaves and branches, and the dry weights of the roots and aerial parts of *Silybum marianum* plants.

ZnO-NPs (mg/L)	Height of plant (cm)	Number of leaves (n)	Number of branches (n)	Dry weight of aerial parts (g)	Dry weight of roots (g)
Control	87.83c*	24.00c	3.833c	52.38d	6.01b
5.0	101.67a	79.33b	4.83c	116.86b	10.20a
10.0	93.83b	79.00b	7.00b	83.78c	7.01b
20.0	100.67a	112.00a	11.33a	130.36a	10.72a

* According to the Tukey–Kramer test, means in a column that are distinguished by the same letter are not statistically different at the 0.05 level of probability.

As shown in **Table 2**, the dry weights of fruits and the number of capitula showed a significant increase under the foliar spraying of ZnO-NPs. A direct relation was interpreted between ZnO-NP concentration and capitula number, as the number gently increased with an increase in ZnO-NP concentration. Higher capitula numbers (11.33) and fruit dry weights (29.49 g) were observed at the 20.0 mg/L concentrations of ZnO-NPs than at the 5.0 and 10.0 mg/L concentrations.

**Table 2:** Effects of different ZnO-NP concentrations on the capitula number and fruit dry weight (in grams) of *Silybum marianum* plants.

ZnO-NPs (mg/L)	Capitula number (n)	Fruit dry weight (g)
Control	5.33 c	8.20 c
5.0	7.83 b	26.54 a
10.0	7.00 b	17.26 b
20.0	11.33 a	29.49 a

* According to the Tukey–Kramer test, means in a column that are distinguished by the same letter are not statistically different at the 0.05 level of probability.

### 3.2 Chemical composition

#### 3.2.1 Photosynthetic pigment content in leaves

The foliar spray of ZnO-NPs at 10.0 mg/L concentration enhanced chlorophyll-*a* (Chl-*a*) and carotenoid contents in *S. marianum* leaves. In contrast, the higher and lower concentrations (5.0 mg/L and 20.0 mg/L) of ZnO-NPs exhibited inhibitory effects on these parameters compared with that in the control treatment. Furthermore, the 5.0 mg/L ZnO-NP concentration showed remarkable enhancing effects on chlorophyll-*b* (Chl-*b*) content compared with that in the control treatment and other ZnO-NP treatments, as shown in **Table 3**.

**Table 3:** Effects of different ZnO-NP concentrations on Chl.-*a*, Chl.-*b*, and carotenoid contents in *Silybum marianum* leaves.

ZnO-NPs (mg/L)	Chl.-*a* (mg/100 g) fresh weight (FW)	Chl.-*b* (mg/100 g) FW	Carotenoids (mg/100 g) FW
Control	25.103 b*	6.804 c	26.07 b
5.0	20.708 c	12.357 a	21.87 c
10.0	33.114 a	10.732 b	33.36 a
20.0	20.030 c	10.276 b	22.86 c

* According to the Tukey–Kramer test, means in a column that are distinguished by the same letter are not statistically different at the 0.05 level of probability.

#### 3.2.2 Mineral element contents in leaves, roots, and fruits

As indicated in **Table 4**, the application of ZnO-NPs at all concentrations (5.0 mg/L, 10.0 mg/L, and 20.0 mg/L) resulted in a significantly reduced N percentage in the leaves of *S. marianum* plants compared with that in plants in the control group, as opposed to an increase in K percentage. Moreover, applying ZnO-NPs at 5.0 mg/L and 10.0 mg/L concentrations resulted in the highest P and Zn percentages in leaves, respectively. All ZnO-NP treatments increased K, N, and P contents in the roots of *S. marianum* plants with statistically significant increases in P and K contents compared with those in the control treatment. Between the three ZnO-NP treatments, the highest P and Zn contents in roots were observed with the 5.0 mg/L concentration, whereas the highest N and K concentrations were observed with the 20.0-mg/L ZnO-NP concentration. Root Zn content increased at both the 5.0-mg/L and 10.0-mg/L ZnO-NP concentrations. However, a reduction in root Zn content was observed in plants treated with the 20.0-mg/L ZnO-NP concentration compared with that in distilled water-treated (control) plants, as shown in **Table 4**.

**Table 4:** Effects of ZnO-NP concentrations on N, P, K, and Zn contents in different parts of *Silybum marianum* plants.

	ZnO-NPs (mg/L)	N (%)	P (%)	K (%)	Zn (%)
Leaves	Control	1.145 a	0.698 ab*	1.73 d	133.69 b*
	5.0	0.755 b	0.779 a	1.85 c	117.45 c
	10.0	0.785 b	0.591 b	1.96 a	146.69 a
	20.0	0.755 b	0.644 ab	1.94 b	127.92 b
Roots	Control	0.84 d	0.794 b	1.76 b	69.32 c
	5.0	0.89 c	0.999 a	2.15 a	98.21 a
	10.0	0.94 b	0.804 ba	2.18 a	71.87 b
	20.0	0.96 a	0.960 a	2.24 a	64.46 c
Fruits	Control	1.90 b	1.073 d	1.645 a	1.645 a
	5.0	2.07 ab	1.187 c	1.650 a	1.665 a
	10.0	2.15 ab	1.215 b	1.665 a	1.520 b
	20.0	2.43 a	1.243 a	1.335 b	1.265 c

* According to the Tukey–Kramer test, means in a column that are distinguished by the same letter are not statistically different at the 0.05 level of probability.

ZnO-NP treatment significantly enhanced N and P contents in fruits compared with that in the fruits of control plants, and this increase in N and P contents increased with increasing ZnO-NP concentrations. The highest ZnO-NP concentration (20.0 mg/L) conferred the greatest enhancements in fruit N and P contents. Both the 5.0- and 10.0-mg/L ZnO-NP concentrations increased the K content in fruits. ZnO-NP treatment at the 20.0-mg/L concentration reduced the K content in fruits compared with that in the control treatment and other ZnO-NP treatments. The highest fruit Zn content was observed at the 5.0-mg/L ZnO-NP concentration, whereas the other ZnO-NP treatments decreased the fruit Zn content compared with that in control plants, as shown in **Table 4**.

**Figure 1:**
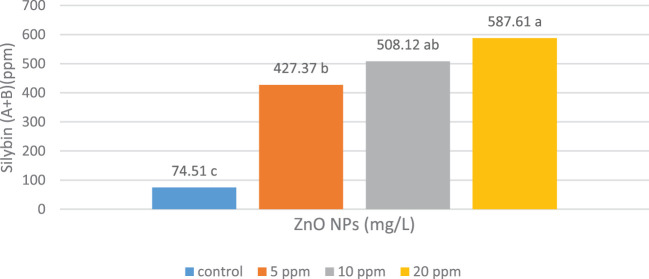
Effects of the foliar spraying of different concentrations of zinc oxide nanoparticles (ZnO-NPs) on silybin (A+B) content in the fruits of *Silybum marianum* plants. * According to the Tukey–Kramer test, means in a column that are distinguished by the same letter are not statistically different at the 0.05 level of probability.

Silybin (A+B) content correlated positively with ZnO-NP concentrations. The treated plants showed differential responses to the three ZnO-NP concentrations with significantly enhanced silybin (A+B) content at all concentrations. The highest silybin (A+B) content (587.6 mg/L) was observed at 20.0 mg/L ZnO-NP concentration, as shown in **Figure 1**.

### 3.3 Effects of ZnO-NP treatments on the expression of *CHS1*, *CHS2*, and *CHS3* genes in the petals of *S. marianum* plants

**Figure 2:**
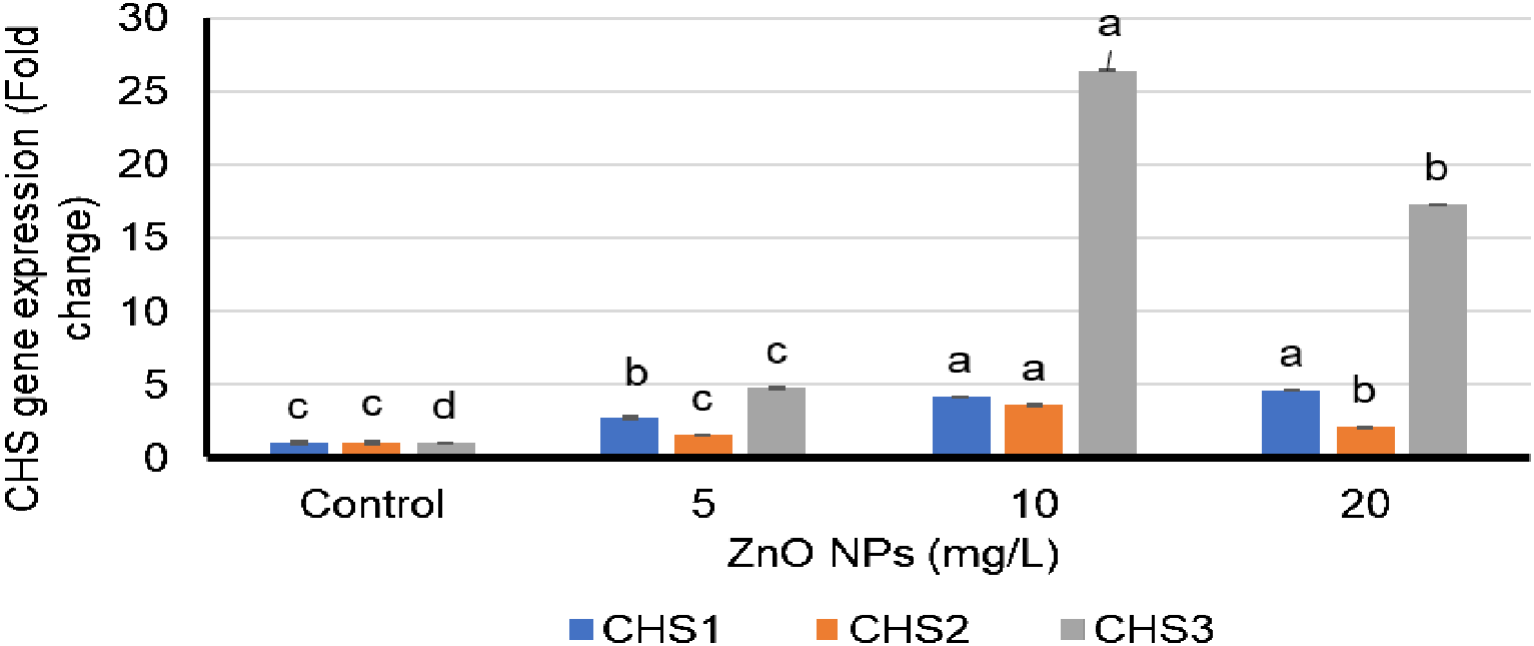
Fold differences in the expression of chalcone synthase (*CHS*) *1*, *2*, and *3* genes in the petals of *Silybum marianum* plants following their foliar spraying treatment with zinc oxide nanoparticles (ZnO-NPs). Means and standard deviations for the individualized treatment groups were calculated based on four biological replicates. For these biological replicates, duplicate polymerase chain reaction was performed. * According to the Tukey–Kramer test, means in a column that are distinguished by the same letter are not statistically different at the 0.05 level of probability.

qPCR was used to study the effects of varying ZnO-NP doses on the expression levels of *CHS1*, *CHS2*, and *CHS3* genes. The expression levels of all three *CHS* genes were elevated by the ZnO-NP foliar spray treatments compared with those in control plants (**Figure 2**). *CHS1* exhibited the highest expression level in the treatment group that received 20 mg/L ZnO-NP foliar spray treatment, followed by the group that received 10 mg/L ZnO-NP foliar spray (**Figure 2**). The highest expression levels of *CHS2* and *CHS3* genes were obtained with the 10 mg/L ZnO-NP foliar spray treatment, followed by the 20 mg/L ZnO-NP foliar spray (**Figure 2**). All ZnO-NP treatment groups showed a consistent relationship between silybin (A+B) content and the expression levels of *CHS* genes (**Figures 1**, **2**).

### 3.4 Effects of *S. marianum* fruit extracts on BMD, BMC, and serum Ca and P levels in rats

**Figure 3:**
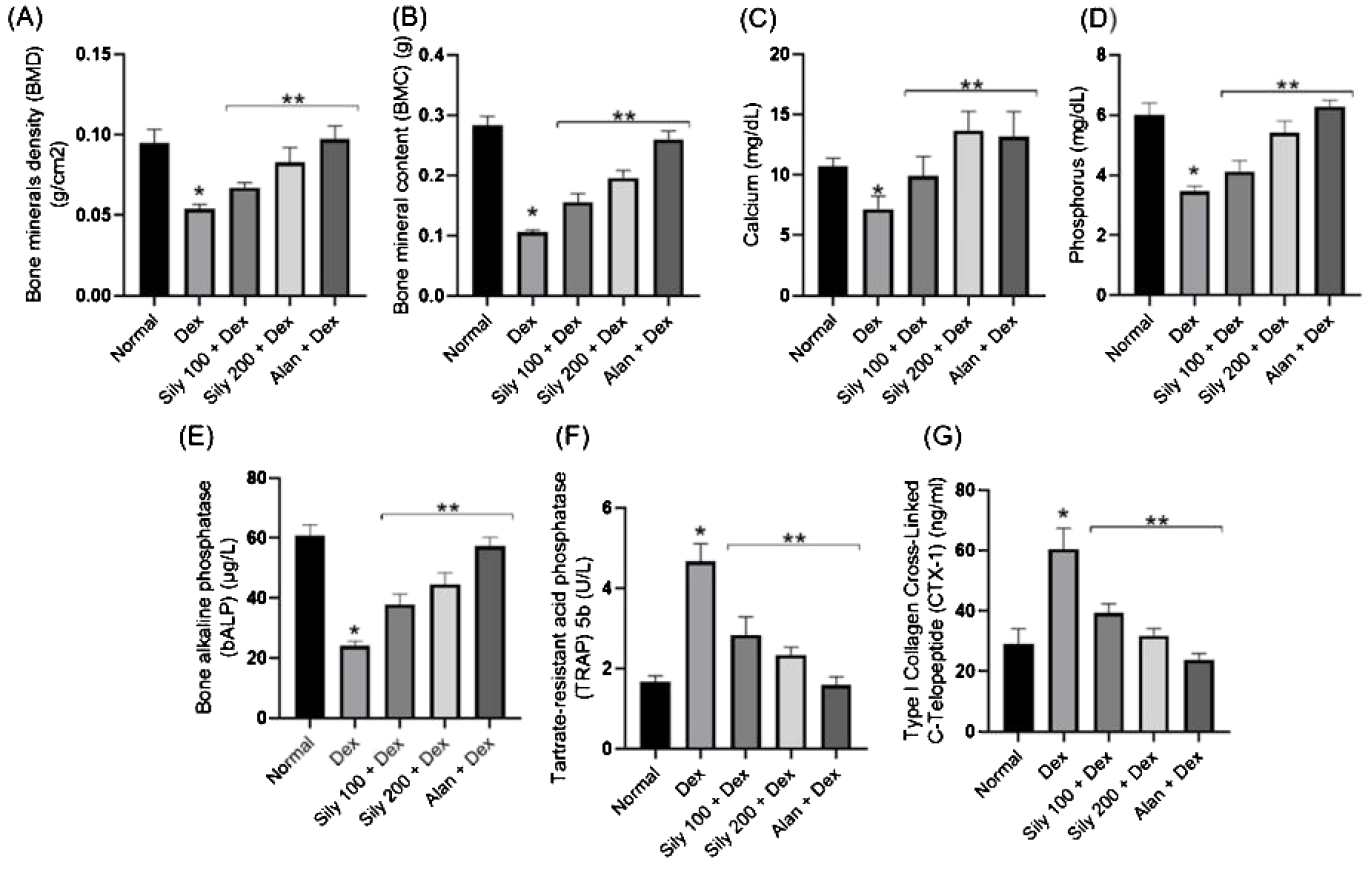
Effects of *Silybum marianum* fruit extracts in a rat model of Dex-induced osteoporosis on **(A)** bone mineral density, **(B)** bone mineral content, and serum **(C)** Ca and **(D)** P levels and on bone resorption markers, including **(E)** bone alkaline phosphatase, **(F)** tartrate-resistant acid phosphatase 5b, and **(G)** carboxy-terminal type I collagen cross-links. Data are expressed as mean ± SD (n = 6). * indicates a significant difference compared with the control group; ** indicates a significant difference (*p <*0.05) compared with the Dex group. Dex, dexamethasone; Sily, *S. marianum* fruit extract; Alen, alendronate.

Dex injections in rats in the Dex group resulted in a significant (*p <*0.05) decrease in their BMD and BMC, which, in turn, decreased their serum Ca and P levels compared with those in rats in the control group. On the contrary, Dex-injected animals treated with *S. marianum* fruit extracts, i.e., those in the Sily-100+Dex and Sily-200+Dex groups, or alendronate (Alen), i.e., those in the Alen+Dex group, displayed a gradual (*p <*0.05) increase in their BMD, BMC, and serum Ca and P levels in comparison with those in animals in the Dex group (Dex alone), as illustrated in **Figures 3A–D**.

### 3.5 Effects of *S. marianum* fruit extracts on the serum levels of bone resorption markers

Dex treatment decreased serum bALP levels compared with that in animals in the control group, whereas cotreatment with Dex and *S. marianum* fruit extracts or Dex and Alen significantly increased serum bALP levels compared with serum bALP levels in animals in the Dex group (**Figure 3E**). The serum levels or activity of bone resorption serum markers, including CTX-I and TRAP5b, were notably higher in animals in the Dex group than in those in the control group, as shown in **Figures 3F, G**.

Notably, the co-administration of either *S. marianum* fruit extracts or Alen with Dex resulted in a significant (*p <*0.05) reduction in serum CTX-I levels and TRAP5b activity compared with that in animals administered Dex alone, as shown in **Figures 3F, G**.

### 3.6 Effects of *S. marianum* fruit extracts on the gene expression levels of osteoclast differentiation markers

**Figure 4:**
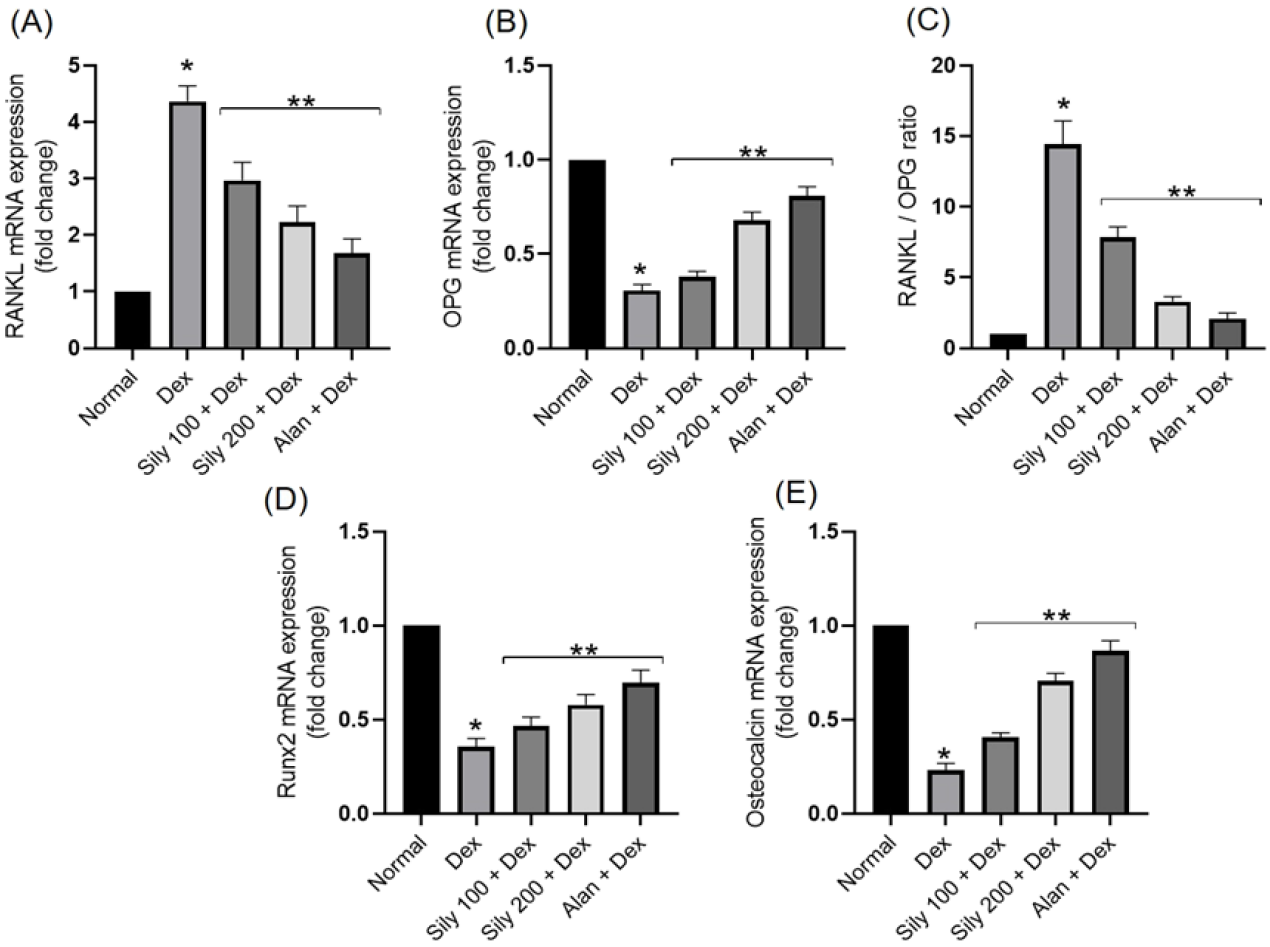
Effects of *Silybum marianum* fruit extracts in a rat model of Dex-induced osteoporosis on the gene expression of **(A)** *RANKL*, **(B)** *OPG*, **(C)** *RANKL*/*OPG* ratio, **(D)** *Runx2*, and **(E)** osteocalcin. Data are presented as mean ± SD (n = 6). * indicates a significant difference (*p <*0.05) compared with the control group; ** indicates a significant difference (*p <*0.05) compared with the Dex group. Dex, dexamethasone; Sily, *S. marianum* fruit extract; Alen, alendronate.

In bone development regulation, various transcriptional regulatory elements and signaling molecules are involved, including Runx2, OPG, and RANKL. Bone hemostasis requires RANKL/OPG signaling regulation. Dex-administered rats exhibited a statistically significant (*p <*0.05) increase in the expression levels of the *RANKL* gene, a negative regulator of osteogenesis, and a statistically significant (*p <*0.05) reduction in the expression levels of the *OPG* gene compared with that in animals in the control group (**Figures 4A, B**). In addition, these rats exhibited a significant increase in their *RANKL*/*OPG* transcript ratio compared with that of animals in the control group, as shown in **Figure 4C**.

Dex administration decreased the mRNA expression levels of osteoblastogenesis regulator gene *Runx2* compared with that in animals in the control group (**Figure 4D**). This reduction in *Runx2* expression levels was significantly (*p <*0.05) reverted following the co-administration of either *S. marianum* fruit extracts or Alen with Dex. Furthermore, OC is considered to be a specific marker of osteoblast function. Dex administration reduced the expression levels of the *OC* gene in comparison with those in animals in the control group, whereas the co-administration of either *S. marianum* fruit extracts or Alen with Dex significantly increased the expression levels of the *OC* gene compared with that in animals administered Dex alone, as shown in **Figure 4E**.

### 3.7 Effects of *S. marianum* fruit extracts on the levels of oxidative stress indicators in femoral bone tissue

**Figure 5:**
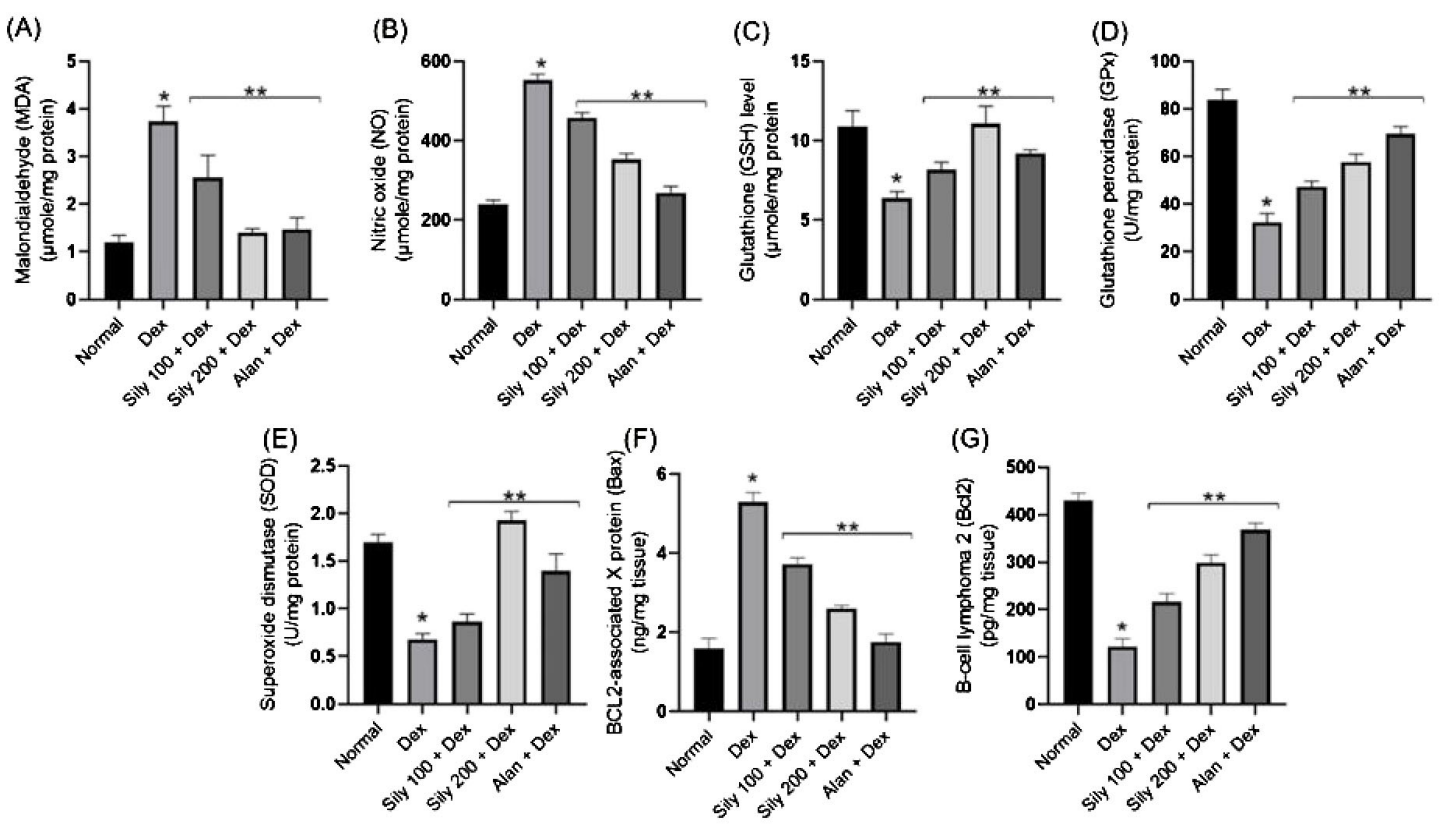
Effects of *Silybum marianum* fruit extracts in a rat model of Dex-induced osteoporosis on oxidative stress indices, including **(A)** MDA, **(B)** NO, **(C)** GSH, **(D)** GPx, and **(E)** SOD and apoptotic markers, including **(F)** BAX and **(G)** BCL2 in bone homogenate. Data are represented as mean ± SD (n = 6). * indicates a significant difference (*p <*0.05) compared with the control group; ** indicates a significant difference (*p <*0.05) compared with the Dex group. Dex, dexamethasone; Sily, *S. marianum* fruit extract; Alen, alendronate.

Dex-induced changes in the levels of oxidative stress indicators were observed in the bone homogenate of animals in the Dex group compared with that in the bone homogenate of animals in the control group (**Figure 5**). The levels of both MDA (lipid peroxidation indicator) and NO increased, accompanied by a significant reduction in bone GSH content and SOD and GPx activities in rats in the Dex group compared with that in rats in the normal group (**Figures 5A–E**). Conversely, the co-administration of either *S. marianum* fruit extracts or Alen with Dex resulted in significant (*p <*0.05) decreases in both MDA and NO levels concurrent with a substantial rise in bone GSH content and SOD and GPx activities compared with that in rats administered Dex alone (**Figures 5A–E**). Notably, the co-administration of *S. marianum* fruit extract Sily-200 resulted in a significant (*p <*0.05) increase in bone GSH content and SOD activity even over the levels observed in animals in the control group, as shown in **Figures 5C, E**.

### 3.8 Effects of *S. marianum* fruit extracts on the levels of apoptosis indicators in femoral bone tissue

Dex administration likely results in a substantial increase in apoptosis in femoral bone tissue, as indicated by a drastic increase in the levels of the apoptosis regulator protein BAX and a reduction in the levels of the anti-apoptotic BCL2 compared with that in animals in the control group. However, these effects were reversed in animals in the Sily100+Dex and Sily-200+Dex groups. Specifically, the co-administration of *S. marianum* fruit extracts at either 100-mg/kg or 200-mg/kg concentrations with Dex resulted in a significant reduction in BAX levels by 30% and 51%, respectively, compared with that in animals administered Dex alone, as shown in **Figure 5F**. Moreover, the co-administration of *S. marianum* fruit extracts with Dex resulted in a significant increase in BCL2 levels compared with that in animals administered Dex alone, as shown in **Figure 5G**.

## 4 Discussion

In this study, we monitored the growth and yield characteristics of *S. marianum* plants; the levels of N, P, K, and Zn in their leaves, roots, and fruits; the levels of photosynthetic pigments in their leaves; and silybin (A+B) content in their fruits following the foliar spraying of ZnO-NPs at various concentrations. The results revealed that the ZnO-NP treatments resulted in concentration-dependent increases in the growth and yield of *S. marianum* plants, with the highest gains observed in plants sprayed with the highest ZnO-NP concentration (20.0 mg/L). Studies have shown that Zn application in plants results in positive growth and yield outcomes in many crops (Awan et al., 2021; Akanbi-Gada et al., 2019; Miliauskienė et al., 2022). ZnO-NPs provide a highly effective supply of Zn because of their NP characteristics, which enable them to enter cells, traverse plant cell membranes, and integrate into metabolic processes (Al Jabri et al., 2022; Faizan et al., 2021). In addition, it offers an alternative to traditional chemical fertilizers by supplying micronutrients necessary for effective plant growth and development, which improves mineral absorption and raises production (Khattab et al., 2023; Adil et al., 2022).

Our findings showed that when an appropriate ZnO-NP concentration (10 mg/L) was applied topically to *S. marianum* plants, the Chl-*a* and carotenoid contents in their leaves increased; however, all three ZnO-NP concentrations enhanced Chl-*b* content in leaves. It has been shown that leaf chlorophyll concentrations increase after applying Zn, which is essential for pigment synthesis, as it functions as an enzyme and protein co-factor as well as a structural and catalytic element (Rudani et al., 2018). The positive effects of ZnO-NPs on leaf chlorophyll content have been documented (Geremew et al., 2023).

In this study, the foliar spraying of ZnO-NPs resulted in concentration-dependent increases in N, P, K, and Zn contents in fruits and roots and P, K, and Zn contents in leaves while lowering N content in leaves. Zn concentrations in the leaves, roots, and fruits of *S. marianum* plants increased with increasing ZnO-NP concentrations. Zn may be efficiently transported via the shoot cortex and translocated via the xylem in *S. marianum* plants, which could account for the variations in Zn concentrations between shoots and roots (Page and Feller, 2015; Claus et al., 2013).

Growth conditions significantly impact various secondary plant products, and stress conditions also affect internal metabolic pathways that lead to the aggregation of correlated natural products (El Sherif et al., 2020). In this study, all three ZnO-NP concentrations significantly increased silybin (A+B) content in *S. marianum* fruits. Numerous plants show increased flavonoid contents following ZnO-NP treatments (Geremew et al., 2023; García-López et al., 2019; Wang et al., 2023; Maher et al., 2023). In this study, silybin (A+B) synthesis in *S. marianum* correlated with the expression of *CHS1*, *CHS2*, and *CHS3* genes, as demonstrated by qPCR amplification and HPLC analyses. In plants, the expression of correlative genes in response to treatments with different plant extracts has been associated with the buildup of important secondary metabolic products, as in *S. marianum* (El Sherif et al., 2023; Sanjari et al., 2015; Alkuwayti et al., 2020).

A skeletal condition called osteoporosis is defined by the loss of mass and the architectural deterioration of bone structures. BMD and BMC are the gold standard tests for identifying osteoporosis. In the present study, Dex administration resulted in a reduction in BMD, BMC, and serum Ca and P levels, aligning with the outcomes of earlier studies (45,46) specifying that inadequate bone mineralization is associated with Dex usage. Dex causes a decline in Ca levels by antagonizing vitamin D, thereby decreasing Ca absorption and renal Ca reabsorption (Hachemi et al., 2018). On the contrary, *S. marianum* fruit extracts increased BMD, BMC, and serum Ca and P levels.

An earlier study examined the effects of the administration of silymarin-rich milk thistle extract (MTE) and its constituent silybin (A+B) on ovariectomy (OVX)-induced osteoporosis in C57BL/6 female mice and revealed that MTE and silybin (A+B) are responsible for improving femoral BMD in mice (Kim et al., 2013).

In the present study, Dex treatment increased the activities of both TRAP5b and acid phosphatase (ACP), which is consistent with prior findings that reported elevated ACP activity and TRAP (an ACP isoenzyme) following Dex treatment (Saleh et al., 2022). TRAP is implicated in bone loss both inside and outside of osteoclasts.

On the contrary, *S. marianum* fruit extracts reduced ACP activity and TRAP levels, which is consistent with the previous finding of Kim et al. (2013), who showed that silymarin-rich MTE and its component silybin (A+B) reduced the TRAP activity of osteoclasts in OVX-induced osteoporotic condition in mice. Additionally, *S. marianum* fruit extracts reversed the reduction in bALP and CTX-I levels triggered by Dex administration.

A previous report showed that silybin (A+B) treatment inhibits bone loss induced by excessive iron intake in the rat model of OVX-induced osteoporosis by promoting the proliferation and differentiation of osteoblasts and, consequently, increasing bALP and CTX-I levels (Tao et al., 2022).

Various signaling molecules and transcriptional regulatory elements, including RANKL, Runx2, and OPG, are implicated in the regulation of osteogenesis. Runx2 is a transcription factor that controls osteoblast differentiation and bone formation. The RANKL/OPG signaling pathway is implicated in bone homeostasis regulation. RANKL/RANK signaling regulates osteoclast formation, activation, and survival in normal bone modeling and remodeling and various pathologic conditions characterized by increased bone turnover. OPG protects bones from excessive resorption by binding to RANKL and preventing it from binding to RANK. Thus, the relative concentrations of RANKL and OPG in bones is a major determinant of bone mass and strength (https://pubmed.ncbi.nlm.nih.gov/18395508/).

In the present study, Dex administration caused a decline in the expression of *OPG* and *Runx2* genes but increased the expression of the *RANKL* gene, thereby increasing the *RANKL*/*OPG* transcript ratio. Although osteocyte maturation limits osteoblastogenesis mediated by GC signaling and enhances bone formation, Runx2 downregulation stimulates osteoblast differentiation (Peng et al., 2021). GCs promote osteoclastogenesis and inhibit anti-osteoclastic cytokine OPG by upregulating RANKL (pro-osteoclastic cytokine) via the RANKL/OPG axis (Di Medio and Brandi, 2021).

On the contrary, *S. marianum* fruit extracts increased the expression of *Runx2* and *OPG* genes and decreased the expression of the *RANKL* gene, thereby decreasing the *RANKL*/*OPG* transcript ratio. An earlier study showed that silymarin is responsible for the stimulation of bALP activity and Ca nodule formation, which has a favorable effect on osteoblast proliferation. Silymarin treatment enhances bone morphogenetic protein (BMP) and Runx2 expression, collagen secretion, *OC* transcription, and SMAD1/5/8 phosphorylation. Kim et al. (2012a, 2012b) revealed that silybin (A+B) is responsible for promoting osteoblastogenesis and osteoprotection in osteoblasts and osteoclasts. The studies reported that by upgrading bone nodule evolution and Ca deposition, silybin (A+B) promotes matrix mineralization and is responsible for the induction of osteoblastogenic biomarkers, including collagen type 1, bone morphogenic protein 2, connective tissue growth factor, ALP, and attenuated RANKL in MC3T3-E1 cells (Kim et al., 2012a). Additionally, silybin (A+B) has a inhibitory effect on RANK transcription and intracellular adhesion molecule-1 expression in RAW 264.7 cells, thereby inhibiting the differentiation of macrophages to multi-nucleated osteoclasts (Kim et al., 2012a). Another study showed that silybin (A+B) inhibits osteoclastogenesis, which is induced by RANKL in RAW264.7 cells by blocking the stimulation of p38 mitogen-activated protein kinase, NF-κB, c-Jun N-terminal kinase (JNK), and extracellular signal-regulated kinase in osteoclast precursors in response to RANKL-mediated activation.

Moreover, silybin (A+B) reduces the increased expression of osteoclast-associated receptor (OSCAR) and nuclear factor of activated T cells (NFAT) c1 during RANKL-induced osteoclastogenesis. The study concluded that silybin (A+B) inhibits osteoclast formation by inhibiting the downstream signaling pathways correlative with RANKL and TNF-α (Kim et al., 2009). Another review described that silybin (A+B) inhibits osteoclast activity and differentiation induced by metastatic prostate cancer cells in RAW264.7 cells by decreasing the DNA binding activity of NFATc1 and its regulators NF-κB and AP1, induced by RANKL, osteoclast-specific markers (cathepsin K, OSCAR, and TRAP), and osteomimicry biomarkers (OC, RANKL, PTHrP, and Runx2) in cell culture (PC3 and C4-2B cells), and helps diagnose PC3 tumors (Mia et al., 2023).

In this study, Dex administration caused an intensification in lipid peroxidation and NO levels, accompanied by a reduction in bone GSH content and GPx and SOD activities. Dex treatment likely causes femur bone tissue to undergo apoptosis, as indicated by a decrease in the expression levels of anti-apoptotic BCL2 protein and an increase in the levels of pro-apoptotic BAX, which is in agreement with the results of Almeida et al. (2011), who showed that GCs increased ROS levels, inducing the activation of a PKCβ/p66shc/JNK signaling pathway that led to the pro-apoptotic effects of Dex on osteoblastic cells.

On the contrary, the administration of *S. marianum* fruit extracts resulted in a decrease in oxidative stress indices, which is consistent with earlier reports that silymarin inhibits oxidative stress in the rat model of OVX-induced osteoporosis, thereby preventing iron overload-induced bone loss, and silybin (A+B) exhibits protective effects in the management of streptozotocin-induced diabetic osteoporosis by limiting oxidative stress (Wang et al., 2017).

*Silybum marianum* fruit extracts significantly reduced BAX levels and increased BCL2 levels, signifying their anti-apoptotic potential. Mao et al. (2018) suggested a novel target of the anti-apoptotic effects of silybin (A+B) in osteoblastic cells, proposing that silybin (A+B) directly decreases the sensitivity of the receptor expression of AGE (RAGE) and downregulates RAGE-mediated mitochondrial pathways, thereby preventing the apoptosis of osteoblastocytes induced by advanced glycation end products.

In summary, silybin (A+B) has been identified to have osteoprotective properties. It is responsible for inhibiting the synthesis of bone-degrading cells (osteoclastogenesis) and promoting the production of bone healing and formation cells (osteoblastogenesis) (Kim et al., 2009, 2012). Silybin (A+B) and silymarin may increase the expression of collagen type I, Runx-2, BMP-2, and ALP, which, in turn, may stimulate osteoblast development in MC3T3-E1 osteoblasts (Kim et al., 2012a, 2012b). BMP signaling can foster osteogenic stem cell differentiation in human bone marrow (Ying et al., 2013).

Silybin (A+B) has antioxidant effects that neutralize free radicals and has anti-apoptotic effects in osteoblasts (Mao et al., 2018). Silybin (A+B) and silymarin reduce RANKL-induced TRAP and cathepsin K induction and cause femoral bone loss in ovariectomized mice by inhibiting osteoclastic development in RAW 264.7 osteoclasts (Kim et al., 2009, 2013).

## 5 Conclusions

Foliar spraying of *S. marianum* plants with ZnO-NP_S_ enhanced their growth and yield and increased silybin (A+B) contents in their leaves. Silybin (A+B) content was strongly associated with the gene expression profiles of *CHS1*, *CH2*, and *CHS3* genes. Furthermore, the elevated content of silybin (A+B) in the extracts of *S. marianum* fruits from plants treated with 20 mg/L ZnO-NP concentrations increased BMD and BMC and decreased the levels of bone resorption markers in Dex-administered rats. The administration of *S. marianum* fruit extracts to Dex-administered rats enhanced in the expression levels of *OPG*, *Runx2*, and *OC* genes as well as a reduction in the expression of the RANKL gene. Thus, *S marianum* fruit extracts protected Dex-administered rats from Dex-induced secondary osteoporosis by promoting osteoblast and bone formation, increasing antioxidant activity, and reducing osteoclast and bone resorption.

## Data Availability

The original contributions presented in the study are included in the article/**Supplementary Material**, further inquiries can be directed to the corresponding author/s.
