# Supplementary material for: Foliar spraying with zinc oxide nanoparticles enhances the anti-osteoporotic efficacy of the fruit extracts of Silybum marianum L. by stimulating silybin production
Source: Front Plant Sci. 2025 Jan 7;15:1421485. doi: 10.3389/fpls.2024.1421485 (PMC11747799; doi:10.3389/fpls.2024.1421485)
Supplement: Supplementary file 1 [file Table1.docx]

**Supplementary Table 1: Chemical properties and compositions of the irrigation water.**

| **Salinity Level (ppm)** | **Cations (meq/L)** | | | | **Anions (meq/L)** | | | | **Sodium Adsorption Ratio (SAR)** |
| --- | --- | --- | --- | --- | --- | --- | --- | --- | --- |
|  | Ca^2+^ | Mg^2+^ | Na^+^ | K^+^ | CO_3_^2−^ | HCO_3_^−^ | SO_4_^2−^ | Cl^−^ |  |
| 864 | 5.72 | 2.02 | 7.27 | 0.38 | 0.28 | 2.68 | 4.03 | 8.4 | 3.43 |

**Supplementary Table 2: Physical and chemical properties of the experimental soil**

| **Characteristic** | **Value** |
| --- | --- |
| Texture | Sandy |
| Sand % | 91.51 |
| Silt % | 5.74 |
| Clay % | 2.75 |
| Saturation % | 23 |
| pH | 7.5 |
| Electrical conductivity (EC) (dS/m) | 2.2 |
| Organic matter (OM) % | 0.05 |
| Total N % | 0.014 |
| Available P ppm | 3.9 |
| Available K ppm | 110 |
